# Biological Effects of Cigarette Smoke in Cultured Human Retinal Pigment Epithelial Cells

**DOI:** 10.1371/journal.pone.0048501

**Published:** 2012-11-14

**Authors:** Alice L. Yu, Kerstin Birke, Johannes Burger, Ulrich Welge-Lussen

**Affiliations:** 1 Department of Ophthalmology, Ludwig-Maximilians-University, Muenchen, Germany; 2 Department of Ophthalmology, Friedrich-Alexander-University, Erlangen, Germany; University of Florida, United States of America

## Abstract

The goal of the present study was to determine whether treatment with cigarette smoke extract (CSE) induces cell loss, cellular senescence, and extracellular matrix (ECM) synthesis in primary human retinal pigment epithelial (RPE) cells. Primary cultured human RPE cells were exposed to 2, 4, 8, and 12% of CSE concentration for 24 hours. Cell loss was detected by cell viability assay. Lipid peroxidation was assessed by loss of *cis*-parinaric acid (PNA) fluorescence. Senescence-associated ß-galactosidase (SA-ß-Gal) activity was detected by histochemical staining. Expression of apolipoprotein J (Apo J), connective tissue growth factor (CTGF), fibronectin, and laminin were examined by real-time PCR, western blot, or ELISA experiments. The results showed that exposure of cells to 12% of CSE concentration induced cell death, while treatment of cells with 2, 4, and 8% CSE increased lipid peroxidation. Exposure to 8% of CSE markedly increased the number of SA-ß-Gal positive cells to up to 82%, and the mRNA expression of Apo J, CTGF, and fibronectin by approximately 3–4 fold. Treatment with 8% of CSE also increased the protein expression of Apo J and CTGF and the secretion of fibronectin and laminin. Thus, treatment with CSE can induce cell loss, senescent changes, and ECM synthesis in primary human RPE cells. It may be speculated that cigarette smoke could be involved in cellular events in RPE cells as seen in age-related macular degeneration.

## Introduction

Age-related macular degeneration (AMD) is the major cause of legal blindness in industrialized nations [1], [2], [3], [4]. It is a multifactorial disease leading to the loss of central vision at the final stage. Both genetic and environmental factors may play a fundamental role in the disease development and progression [5], [6]. Cigarette smoke is the single most important environmental risk factor for AMD [7], [8], [9]. Recent studies have detected a two- to threefold increased risk for AMD in smokers compared to non-smokers [8], [10]. One reason for these adverse effects could be attributed to the various potent oxidants, which are contained in cigarette smoke [11], [12]. Therefore, it is assumed that cigarette smoke mediates its toxic effects via increased production of reactive oxygen species (ROS) and thus oxidative stress [10].

Oxidative stress plays the key role in the process of ageing and age-related diseases. In age-related diseases, it induces a number of biological events such as cell death, advanced senescence, and extracellular matrix (ECM) production [13]. These characteristic findings can also be found in ocular age-related diseases such as AMD. In the setting of massive deposition of extracellular debris, the loss of retinal pigment epithelial (RPE) cells is the key event of the atrophic form of AMD leading to the loss of central vision [14]. In early AMD, advanced cellular senescence of the RPE may represent an initial step in the pathogenesis of AMD [15]. Cellular senescence can be identified by increased senescence-associated ß-galactosidase (SA-ß-Gal) activity and elevated expression of senescence-associated biomarkers such as apolipoprotein J (Apo J), connective tissue growth factor (CTGF), and fibronectin [16], [17], [18]. ApoJ, also called clusterin, is abundant in drusen [19]. CTGF and fibronectin accumulate in the Bruch's membrane, in drusen and in basal linear deposits of AMD eyes [20], [21], [22]. Fibronectin and also the basement membrane component laminin have been shown to be secreted by senescent human RPE cells [23]. In AMD donor eyes, increased ECM accumulation can lead to a diffuse thickening of the Bruch's membrane beneath the RPE, and thus an impaired diffusion of oxygen towards the retina [23], [24].

In this study, we hypothesized that cigarette smoke is responsible for these cellular changes in the RPE of AMD patients. In our experiments, we used cigarette smoke extract (CSE) as a well-established *in vitro* model of cigarette smoke exposure [25], [26], [27]. We first examined at which concentration CSE could induce cell death in primary cultured human RPE cells. Furthermore, we wanted to known whether or not CSE could increase lipid peroxidation in human RPE cells. In addition, we investigated the effects of CSE on senescence-associated changes and the synthesis of ECM components. These data should reveal further information about the potential role of cigarette smoke in cellular events of AMD.

## Materials and Methods

### Isolation of human RPE cells

For the total study, five human donor eyes were obtained from the eye bank of the Ludwig-Maximilians-University, Munich, Germany, and were processed within 4 to 16 hours after death. The donors ranged in age between 30 and 43 years. None of the donors had a history of eye disease. Methods of securing human tissue were humane, included proper consent and approval, complied with the declaration of Helsinki, and was approved by the Department of Medicine of the Ludwig-Maximilians-University, Munich. The consent statement was written. Human retinal pigment epithelial (RPE) cells were harvested following the procedure as described previously [28], [29], [30]. In brief, whole eyes were thoroughly cleansed in 0.9% NaCl solution, immersed in 5% polyvinylpyrrolidone iodine (Jodobac; Bode-Chemie, Hamburg, Germany), and rinsed again in NaCl solution. The anterior segment from each donor eye was removed, and the posterior poles were examined with the aid of a binocular stereomicroscope to confirm the absence of gross retinal disease. Next, the neural retinas were carefully peeled away from the RPE-choroid-sclera using fine forceps. The eyecup was rinsed with Ca^2+^ and Mg^2+^ -free Hank's balanced salt solution, and treated with 0.25% trypsin (GIBCO, Karlsruhe, Germany) for 1 hour at 37°C. The trypsin was aspirated and replaced with Dulbecco's modified Eagles medium (DMEM, Biochrom, Berlin, Germany) supplemented with 20% fetal calf serum (FCS) (Biochrom). Using a pipette, the media was gently agitated, releasing the RPE into the media by avoiding damage to Bruch's membrane.

### Human RPE cell culture

The human RPE cell suspension was added to a 50 ml flask (Falcon, Wiesbaden, Germany) containing 20 ml of DMEM supplemented with 20% FCS and maintained at 37°C and 5% CO_2_. Epithelial origin was confirmed by immunohistochemical staining for cytokeratin using a pan-cytokeratin antibody (Sigma-Aldrich, Deisenhofen, Germany) [31]. RPE cells were characterized by positive immunostaining with RPE65-antibody, a RPE-specific marker (anti-RPE65, Abcam, Cambridge, UK), and quantified by flow cytometry showing that nearly 100% of cells were RPE65 positive in each cell culture. The cells were tested and found free of contaminating macrophages (anti-CD11, Sigma-Aldrich) and endothelial cells (anti-von Willbrand factor, Sigma-Aldrich). The expression of zonula occludens-1 (ZO-1; Molecular Probes, Darmstadt, Germany) was used as a marker of RPE tight junctions. After reaching confluence, primary RPE cells were subcultured and maintained in DMEM supplemented with 10% FCS at 37°C and in 5% CO_2_. Confluent primary RPE cells of passage 3 to 5 were exposed to cigarette smoke extract (CSE) in a concentration from 2, 4, 8 and 12% for 24 hours.

To generate aqueous CSE, the smoke of commercially available filter cigarettes (Marlboro, Philip Morris GmbH, Berlin, Germany; nicotine: 0.8 mg; tar: 10 mg) was bubbled through 25 ml pre-warmed (37°C) serum-free DMEM as described in Bernhard et al. [26]. The cigarettes were syringe-smoked in a similar apparatus as described by Carp and Janoff [32] at a rate of 35 ml/2 sec followed by a pause of 28 sec. This rate of smoking should simulate the smoking habits of an average smoker [33]. The resulting suspension was adjusted to pH 7.4 with concentrated NaOH and then filtered through a 0.22-µM-pore filter (BD biosciences filter Heidelberg, Germany) to remove bacteria and large particles. This solution, considered to be 100% CSE, was applied to RPE cultures within 30 min of preparation. CSE concentrations in the current study ranged from 2 to 12%. CSE preparation was standardized by measuring the absorbance (OD, 0.86±0.05) at a wavelength of 320 nm. The pattern of absorbance (spectrogram) observed at λ_320_ showed insignificant variation between different preparations of CSE. The nicotine in the CSE was determined by high-performance liquid chromatography with ultraviolet detection and resulted in 47.1 ng nicotine/ ml cigarette smoke on average. This concentration was similar to the plasma nicotine concentration of an average smoker [43.7 ng/ml+/−38] [34]. After exposure to CSE, cells were kept for 72 hours under serum free conditions. For control experiments, air was bubbled through the serum-free DMEM, pH was adjusted to 7.4, and sterile filtered as described earlier. The medium was changed at the same time points.

### Cell viability assay

Cell viability was quantified based on a two-colour fluorescence assay, in which the nuclei of non-viable cells appear red because of staining by the membrane-impermeable dye propidium iodide (Sigma-Aldrich), whereas the nuclei of all cells were stained with the membrane-permeable dye Hoechst 33342 (Intergen, Purchase, NY). Confluent cultures of RPE cells growing on coverslips in four well tissue culture plates were either non-stressed or exposed to CSE. For evaluation of cell viability, cells were washed in PBS and incubated with 2.0 µg/ml propidium iodide and 1.0 µg/ml Hoechst 33342 for 20 minutes at 37°C. Subsequently, cells were analyzed with a fluorescence microscope (Leica DMR, Leica Microsystems, Wetzlar, Germany). Representative areas were documented with Leica IM 1000 software (Leica Microsystems, Heerbrugg, Switzerland), with three to five documented representative fields per well. The labelled nuclei were then counted in fluorescence photomicrographs, and dead cells were expressed as a percentage of total nuclei in the field. All experiments were run in triplicate in RPE cultures from three donors and repeated three times.

### Assessment of lipid peroxidation

Oxidative stress can be assessed by markers of lipid peroxidation. A sensitive and specific assay for lipid peroxidation is based on metabolic incorporation of the fluorescent oxidation-sensitive fatty acid, *cis*-parinaric acid (PNA), a natural 18-carbon fatty acid with four conjugated double bonds, into membrane phospholipids of cells [35], [36]. Oxidation of PNA results in disruption of the conjugated double bond system that cannot be re-synthesized in mammalian cells. Therefore, lipid peroxidation was estimated by measuring loss of PNA fluorescence. Briefly, treated cells were incubated with 10 µM PNA (Molecular Probes, Invitrogen, UK) at 37°C for 30 minutes in the dark. The media was then removed and cells washed three times with phosphate-buffered saline (PBS). Afterwards, cells were scraped into 2 ml PBS using a rubber policeman. The suspension was then added to a fluorescence cuvette and measured at 312-nm excitation and 455-nm emission. A blank (unlabelled cells) was measured and subtracted from all readings. This method has been validated by treating the RPE cultures with different concentrations of hydrogen peroxide. A dose-dependent loss of fluorescence could be observed (data not shown). All experiments were run in triplicate in RPE cultures from three donors and repeated three times.

### Senescence-associated ß-galactosidase activity

The proportion of RPE cells positive for the senescence-associated ß-galactosidase (SA-ß-Gal) activity was determined as described by Dimri et al. [37]. Briefly, treated RPE cells were washed twice with PBS and fixed with 2% formaldehyde and 0.2% glutaraldehyde in PBS at pH 6.0 at room temperature (RT) for 4 minutes. Cells were then washed twice with PBS and incubated under light protection for 8 hours at 37°C with fresh SA-ß-Gal staining solution (1 mg/ml 5-bromo-4-chloro-3-indoyl-ß-D-galactopyranoside (X-gal), 40 mM citric acid/sodium phosphate, pH 6.0, 5 mM potassium ferrocyanide, 5 mM potassium ferricyanide, 150 mM NaCl, 2 mM MgCl_2_ diluted in PBS). Cells were then examined for the development of blue color and photographed at low magnification (200×) using a light microscope. All experiments were run in triplicate in RPE cultures from three donors and repeated three times.

### RNA isolation and real-time PCR

Total RNA was isolated from 10 mm petri dishes by the guanidium thiocyanate-phenol-chloroform extraction method (Stratagene, Heidelberg, Germany). Structural integrity of the RNA samples was confirmed by electrophoresis in 1% Tris-acetate-EDTA (TAE)-agarose gels. Yield and purity were determined photometrically. After RNA isolation, mRNA was transcribed to cDNA via reverse transcriptase. This cDNA was then used for specific real-time PCR. Quantification of human mRNA was performed with specific primers during 40 cycles with a LightCycler Instrument (LightCycler System, Roche Diagnostics, Mannheim, Germany). The primers selected were apolipoprotein J (Apo J) forward primer 5′- ggacatccacttccacagc -3′ and reverse primer 5′- ggtcatcgtcgccttctc -3′; connective tissue growth factor (CTGF) forward primer 5′- ctgcaggctagagaagcagag -3′ and reverse primer 5′- gatgcactttttgcccttct -3′; fibronectin forward primer 5′-ctggccgaaaatacattgtaaa-3′ and reverse primer 5′-ccacagtcgggtcaggag-3′; and GAPDH forward primer 5′-agccacatcgctcagacac-3′ and reverse primer 5′-gcccaatacgaccaaatcc-3′. Primers and probes were found with the programme ProbeFinder Version: 2.04. The standard curve was obtained from probes of three different untreated human RPE cell cultures. To normalize differences of the amount of total RNA added to each reaction, GAPDH was simultaneously processed in the same sample as an internal control. The level of Apo J, CTGF and fibronectin mRNA was determined as the relative ratio (RR), which was calculated by dividing the level of Apo J, CTGF and fibronectin mRNA by the level of the GAPDH housekeeping gene in the same samples. All experiments were run in triplicate in RPE cultures from three donors and repeated three times.

### Protein extraction and western blot analysis

For nuclear extracts, cells were washed twice with ice-cold PBS, collected, and lysed in three times packed cell volumes of low-salt hypotonic cell lysis buffer [20 mM HEPES pH 7.5, 10 mM KCl, 5 mM MgCl2, 0.5 mM EDTA, 0.1% TritonX-100, 10% glycerol, protease inhibitor cocktail (Roche)] for 10 min on ice. After centrifugation (19,000 *g* for 30 minutes at 4°C) in a microfuge, the supernatants were transferred to fresh tubes and stored at −70°C for future use. The protein content was measured by the bicinchoninic acid (BCA) protein assay (Pierce, Rockford, IL). Denatured proteins (2 µg) were separated under reducing conditions by electrophoresis using 10% SDS-polyacrylamide gels. Thereafter, the proteins were transferred with tank blotting onto a nitrocellulose membrane (Protran Ba-183; Whatman, Dassel, Germany) and probed with a mouse monoclonal anti-human ApoJ antibody (Abcam) and rabbit polyclonal anti-human CTGF antibody (Abcam) as described previously [38]. These antibodies were used at a dilution of 1∶1000, respectively. Secondary alkaline phosphatase (AP)-conjugated goat anti-mouse IgG (Sigma-Aldrich) or AP-conjugated goat anti-rabbit IgG antibodies (Sigma-Aldrich) were incubated for 30 minutes at a dilution of 1∶2500 at room temperature. After substrate incubation (CDP-star; Roche) the signals were visualized by exposure to light sensitive films (Hyperfilm ECL; GE Healthcare, Munich, Germany), which were digitized and densitometrically quantified with the Multi Gauge V3.1 software (Fujifilm, Duesseldorf, Germany). All experiments were run in triplicate with three different RPE cultures from three donors.

### Analysis of fibronectin and laminin secretion into culture media

Release of fibronectin and laminin into culture media of RPE cells was measured using corresponding QuantiMatrixTM Human Fibronektin ELISA kits (Millipore, Billerica, MA, USA) and QuantiMatrixTM Human Laminin ELISA kits (Millipore) according to the manufacturer's instructions. All experiments were run in triplicate with three different RPE cultures from three donors.

### Statistical analysis

Results for the analyses of RPE cell death, lipid peroxidation, SA-ß-Gal activity, real-time PCR, western blot and ELISA experiments are expressed as the mean ± s.d. For comparison of means between two groups, an unpaired t-test was employed. Statistical significance was defined as P<0.05.

## Results

### ZO-1 expression in cultured human RPE cells

**Figure 1:**
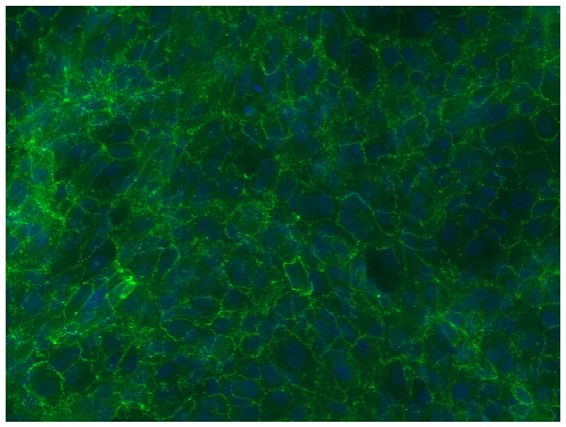
ZO-1 immunofluorescence staining in primary human RPE cultures. DAPI nuclear counterstaining.

The expression and localization of ZO-1 was used to define the tight junction structure of the cultured human RPE cells. Each RPE cell was outlined by the expression of ZO-1 (Figure 1).

### Cigarette smoke extract induced cell death

**Figure 2:**
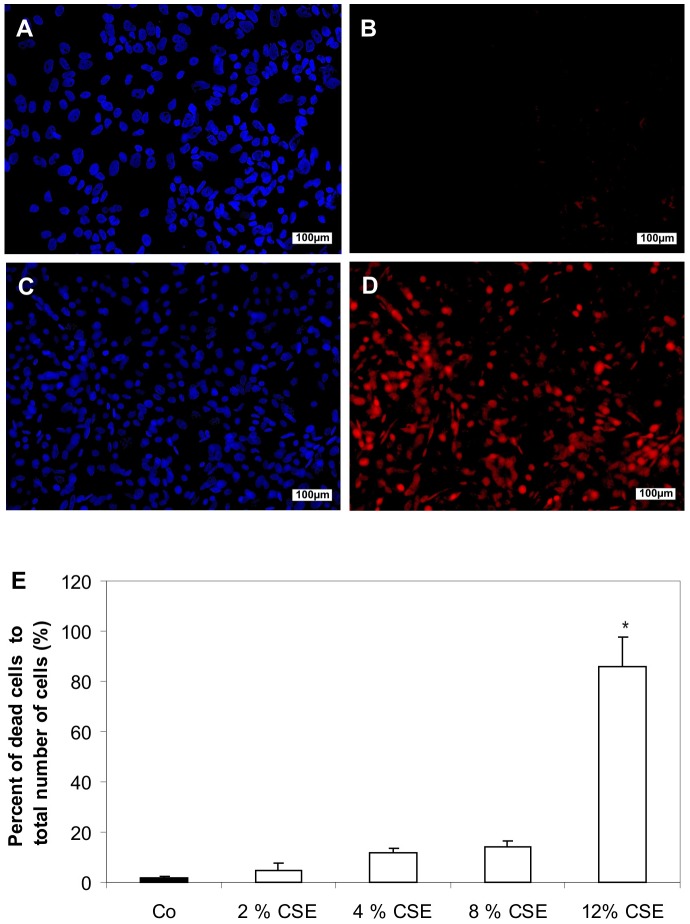
CSE induced cell death detected by live dead assay. Representative fluorescence photomicrographs of Hoechst 33342-stained RPE cells in (**A**) untreated controls or (**C**) cells treated with 12% of CSE for 24 hours. Scale bar: 100 µm. (**B, D**) Non-viable cells in the corresponding field. Scale bar: 100 µm. (**E**) Quantification of the number of non-viable cells. The percentage of dead cells was scored by counting at least 700 cells in fluorescence photomicrographs of representative fields. Data presented as a mean ± s.d. of nine experiments with three different cell cultures from different donors (*P<0.05). Co, control.

To determine the cytotoxic effects of cigarette smoke extract (CSE), primary cultured human retinal pigment epithelial (RPE) cells were treated with 2, 4, 8 and 12% of CSE (Fig. 2). In this cell viability assay, untreated control cells demonstrated almost no dead cells staining red by propidium iodide (Fig. 2B). Incubation of cultured human RPE cells with 2, 4, and 8% of CSE led to elevated proportions of non-viable cells with 5.1+/−2.4%, 12.0+/−1.7%, and 14.0+/−2.4% of total cells (Fig. 2E). The most pronounced effect was seen after treatment with 12% of CSE, which significantly increased the proportion of non-viable RPE cells to 86.2+/−11.4% of total cells (Figs. 2D, 2E). Based on these results, only concentrations of 2, 4, and 8% of CSE were used in the subsequent experiments.

### Cigarette smoke extract increased lipid peroxidation

**Figure 3:**
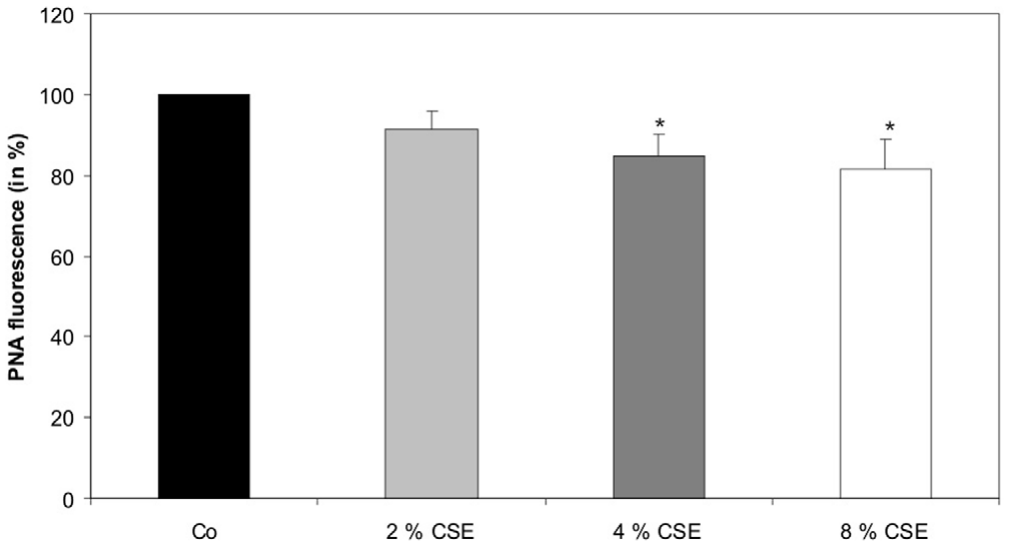
CSE increased lipid peroxidation. *cis*-parinaric acid (PNA) fluorescence was analysed after 2, 4, and 8% concentration of CSE for 24 hours. Data are expressed as the percentage of PNA fluorescence of untreated control cells kept for 24 hours and represent the mean ± s.d of results of nine experiments with three different cell cultures from different donors (*P<0.05). Co, control.

Lipid peroxidation of the cytoplasm membrane of primary cultured human RPE cells was assessed by increased loss of *cis*-parinaric acid (PNA) fluorescence (Fig. 3). The PNA fluorescence of untreated cells was set to 100%. We could observe a decrease of PNA fluorescence after treatment of RPE cells with 2 and 4% concentration of CSE for 24 hours to 91.3+/−4.7% and 84.7+/−5.3% as compared to untreated control cells. The most significant decrease of PNA fluorescence to 81.7+/−7.3% was observed after exposure of RPE cells to 8% of CSE (Fig. 3).

### Cigarette smoke extract induced SA-ß-Gal activity

**Figure 4:**
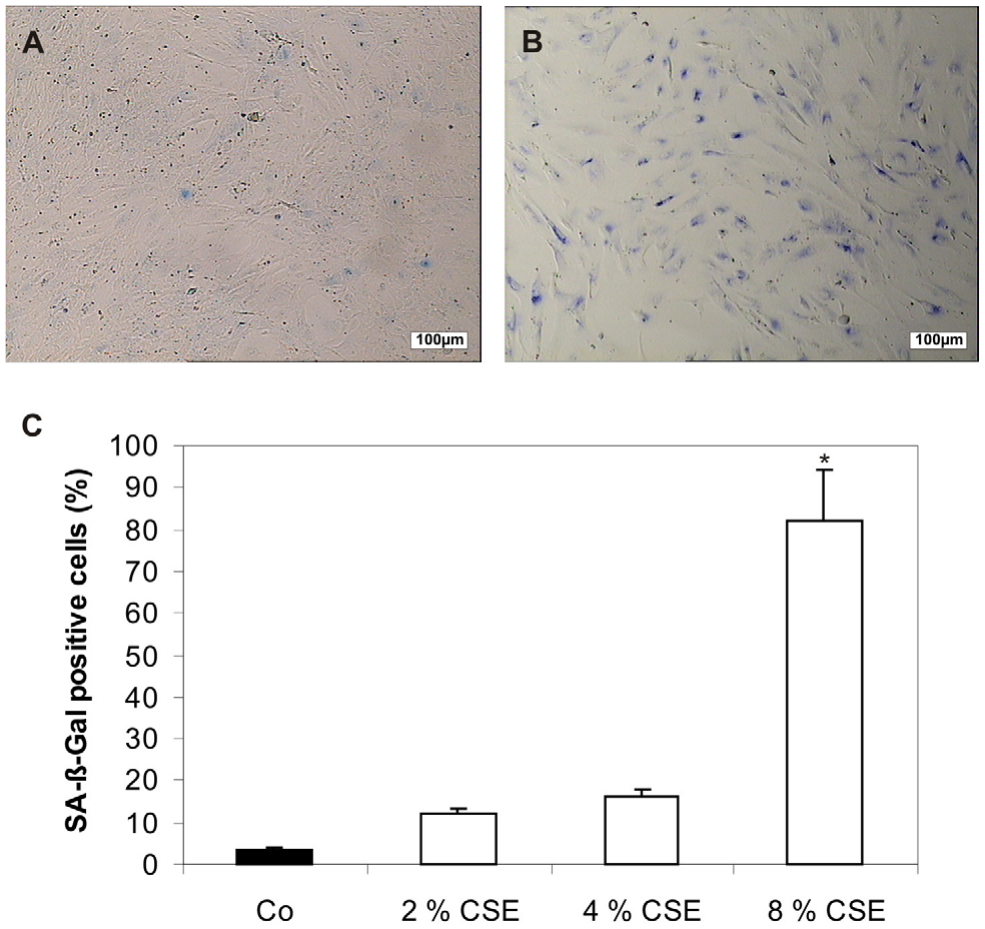
CSE induced SA-ß-Gal activity in cultured human RPE cells. (**A**) Morphology and SA-ß-Gal activity of untreated human RPE cells. Only single cells were stained blue indicating SA-ß-Gal activity. Scale bar: 100 µm. (**B**) In contrast, RPE cells of the same passage exposed to 8% of CSE showed a marked increase of SA-ß-Gal activity. Scale bar: 100 µm. (**C**) Quantification of the number of SA-ß-Gal positive cells. The percentage of SA- ß-Gal activity was analyzed after exposure to 2, 4, and 8% of CSE and scored by counting at least 300 cells in phase contrast photomicrographs of representative fields. Data (mean ± s.d.) are based on the sampling of 6 to 10 photomicrographs per condition from nine experiments with three different cell cultures from different donors (*P<0.05). Co, control.

Human RPE cells were treated for 2, 4, and 8% concentration of CSE for 24 hours (Fig. 4). Untreated control cells showed 3.5+/−0.6% of senescence-associated ß-Galactosidase (SA-ß-Gal) positive RPE cells (Figs. 4A, 4C). Exposure to 2% and 4% of CSE increased the number of SA-ß-Gal positive RPE cells to 12.0+/−1.4% and 16.0+/−1.7% of all treated cells (Fig. 4C). The most pronounced effect was observed after exposure of cells to 8% of CSE with a proportion of 82.0+/−12.0% SA-ß-Gal positive cells (Figs. 4B, 4C). We have not observed any differences in SA-ß-Gal staining in RPE cell cultures from different donors (data not shown).

### Cigarette smoke extract induced mRNA expression of Apo J, CTGF, and fibronectin

**Figure 5:**
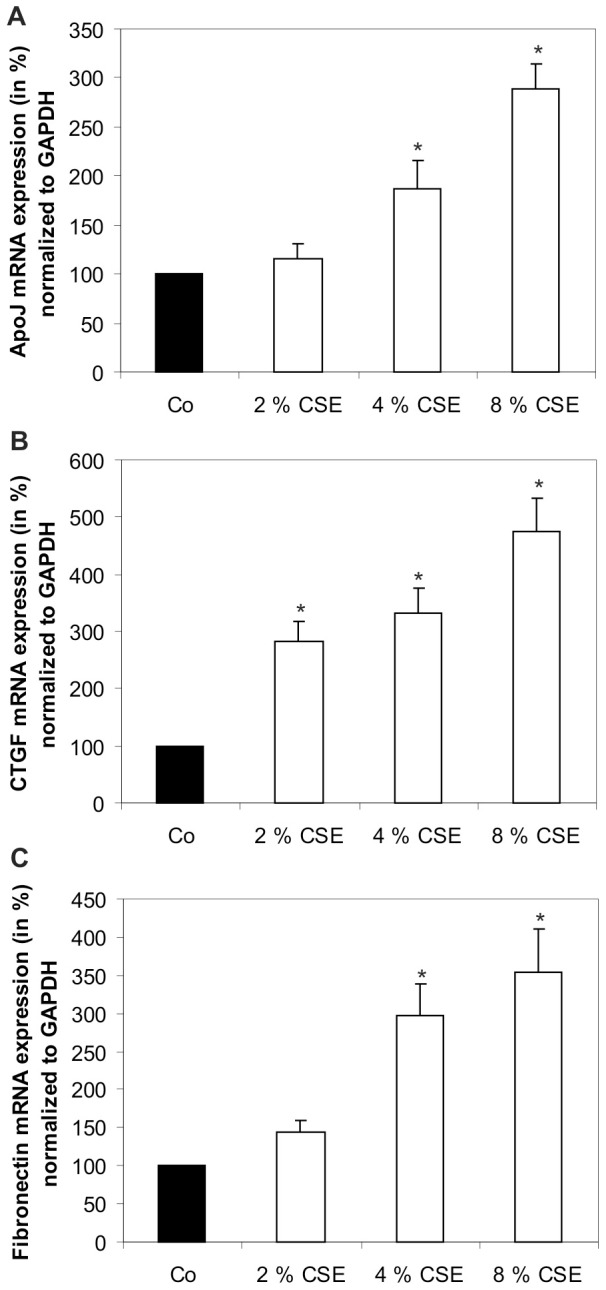
CSE increased Apo J, CTGF, fibronectin mRNA expression. mRNA expression of (**A**) Apo J, (**B**) CTGF, (**C**) fibronectin. Real-time PCR analysis was conducted after treatment with 2, 4, and 8% of CSE. Results were normalized to GAPDH as reference. The steady-state mRNA levels of these senescence-associated genes in untreated control cells were set to 100%. Results are given as mean ± s.d. of nine experiments with three different cell cultures from different donors (*P<0.05). Co, control.

The mRNA expressions of apolipoprotein J (Apo J), connective tissue growth factor (CTGF), and fibronectin were detected by real-time PCR analysis. The signals generated in untreated control cells were set to 100% (Figs. 5A, 5B, 5C). Expressions of Apo J (Fig. 5A), CTGF (Fig. 5B), and fibronectin (Fig. 5C) were measured after treatment with 2, 4, and 8% of CSE. Exposure to 2% and 4% of CSE increased the expression of Apo J to 1.2+/−0.2 fold and 1.9+/−0.3 fold, the expression of CTGF to 2.8+/−0.4 fold and 3.3+/−0.4 fold, and the expression of fibronectin to 1.5+/−0.2 fold and 3.0+/−0.4 fold, as compared to untreated control cells. The most significant effects were seen after exposure to 8% of CSE. In these cells, the Apo J mRNA expression increased by 2.9+/−0.3 fold (Fig. 5A), the CTGF expression by 4.8+/−0.6 fold (Fig. 5B), and the fibronectin expression by 3.5+/−0.6 fold (Fig. 5C), as compared to untreated control cells.

### Cigarette smoke extract induced protein expression of Apo J and CTGF

**Figure 6:**
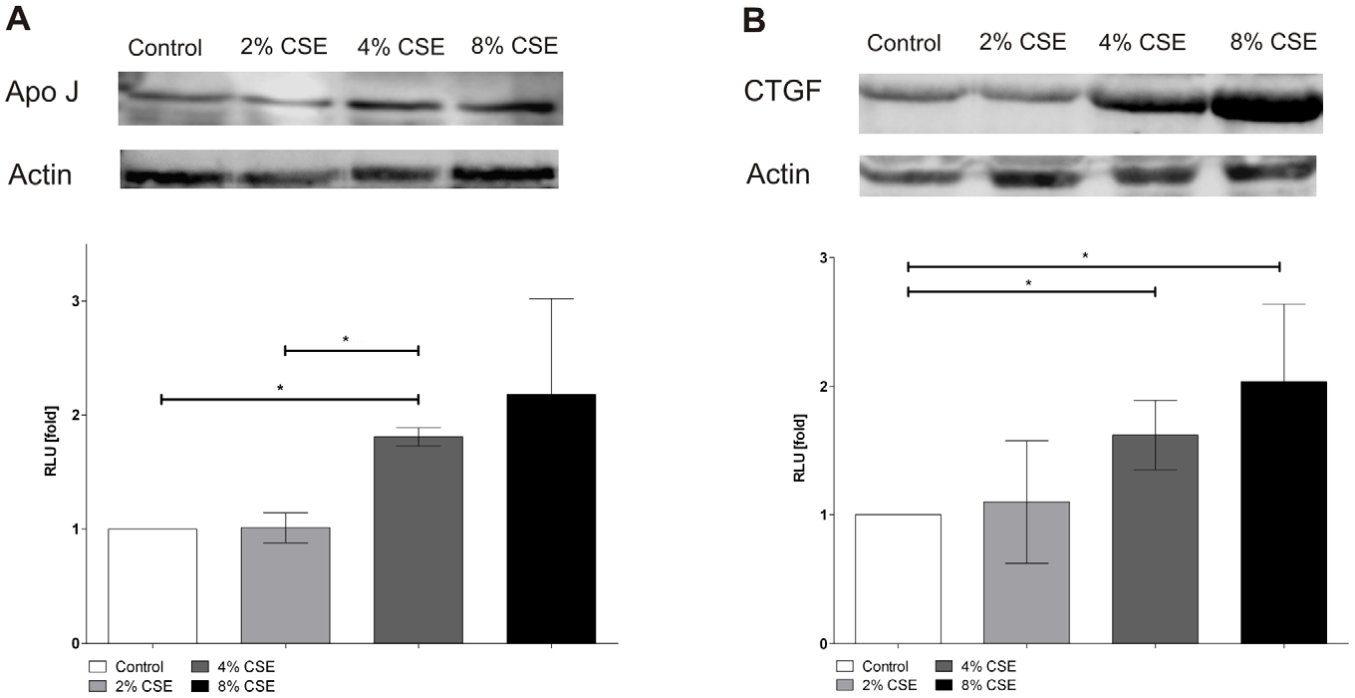
CSE increased Apo J, CTGF protein expression. Protein expression of (**A**) Apo J, (**B**) CTGF. Data are expressed as x-fold changes compared to the signals of untreated control cells and represent the mean ± s.d. of results of three experiments with three different cell cultures from different donors (*P<0.05).

The protein expression of Apo J and CTGF was analysed by western blot analysis. Data are expressed as x-fold changes compared to the signals of untreated control cells (Figure 6). Protein expressions of Apo J and CTGF were measured after treatment with 2, 4, and 8% of CSE. There was a marked increase of Apo J protein expression after treatment of cultured human RPE cells with 4 and 8% of CSE as compared to untreated control cells (2% CSE: 1.0±0.1 fold; 4% CSE: 1.8±0.1 fold; 8% CSE: 2.2±0.8 fold) (Figure 6A). Similarly, CTGF protein expression was significantly elevated after exposure to 4 and 8% of CSE compared to untreated control cells (2% CSE: 1.1±0.5 fold; 4% CSE: 1.6±0.3 fold; 8% CSE: 2.0±0.6 fold) (Figure 6B).

### Cigarette smoke extract induced fibronectin and laminin secretion

**Figure 7:**
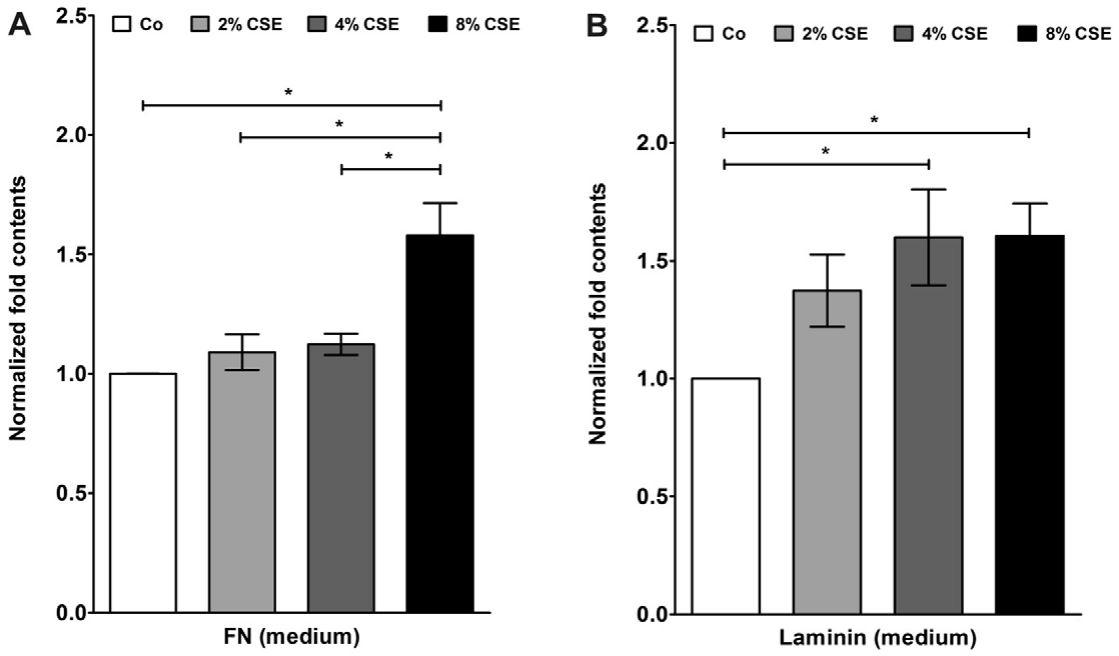
CSE increased fibronectin, laminin protein secretion. Protein secretion of (**A**) fibronectin (FN) and (**B**) laminin into culture media. Error bars: ± s.d. of results from three experiments with three different cell cultures (*P<0.05). Co, control.

To determine the fibronectin and laminin secretion of cultured human RPE cells by CSE exposure, we have used commercially available ELISA assays. Data are expressed as x-fold changes compared to the basal secretion levels of untreated control cells (Figure 7). Treatment of human RPE cells with 2, 4 and 8% of CSE increased the fibronectin secretion by 1.1±0.1 fold, 1.1±0.1 fold and 1.6±0.2 fold, as compared to untreated control cells. Furthermore, exposure of RPE cells to 2, 4 and 8% of CSE also led to increased levels of laminin secretion by 1.4±0.3 fold, 1.6±0.4 fold and 1.6±0.2 fold, compared to untreated control cells (Figure 7).

## Discussion

Previous epidemiological studies have demonstrated that cigarette smoking significantly increases the risk of age-related macular degeneration (AMD) [7], [8], [9]. However, the impact of cigarette smoke on pathogenic processes of AMD is still unknown. One reason for the harmful effects of cigarette smoke on human cells is the generation of reactive oxygen species (ROS) and therefore oxidative stress [10]. Oxidative stress is also an important risk factor for ocular age-related diseases such as AMD. The loss of retinal pigment epithelial (RPE) cells is the major characteristic event of the atrophic form of AMD [39]. Previous *in vitro* studies have already demonstrated cytotoxic effects of cigarette smoke [40], [41]. Cigarette smoke is known to contain an abundant number of toxic compounds. In ARPE-19 cells, specific toxic elements of cigarette smoke such as acrolein and benzopyrene may lead to reduced cell viability [40], [41]. Cadmium, which is found in higher amounts in retinal tissues of AMD eyes, is also released from cigarette smoke and can induce RPE cell death [42]. In our experiments, treatment of primary human RPE cells with 2, 4, and 8% of cigarette smoke extract (CSE) had no significant effects on RPE cell loss. However, exposure of cells to 12% of CSE markedly induced RPE cell death. At the first glance, these results are in contrast to previous investigations with ARPE-19 cells, which showed a decreased viability after 0.5% of CSE [43]. However, it must be taken into account that in Bertram et al. [43], CSE was generated by the smoke of research-grade cigarettes (Kentucky Tobacco Research Council, Lexington, KY, U.S.A.), which contain a much higher nicotine concentration than commercially available filter cigarettes. Therefore, CSE may be toxic for RPE cells at higher concentrations. Interestingly, Patil et al. [44] did not find decreased cell viability of human ARPE-19 cells after treatment with nicotine itself. This observation may be explained by the fact that not only nicotine itself but also other toxic elements of cigarette smoke influence the RPE viability. Furthermore, in our subsequent experiments, treatment of primary human RPE cells with 2, 4, and 8% of CSE increased lipid peroxidation estimated by the loss of *cis*-parinaric acid (PNA) fluorescence. These results suggest that lower concentrations of CSE can induce the release of ROS and thus cause oxidative stress in primary human RPE cells.

At the cellular level, oxidative stress can trigger the so-called ‘stress-induced premature senescence’ (SIPS) [15], [45]. There is a growing body of evidence suggesting that RPE cells also undergo an accelerated ageing process in AMD [24], [46], [47], [48]. We have previously shown that sublethal concentrations of hydrogen peroxide induced senescence-associated ß-Galactosidase (SA-ß-Gal) activity in primary cultured RPE cells [29]. In the experiments of the current study, treatment of primary human RPE cultures with CSE could significantly increase the proportion of SA-ß-Gal positive cells. Positive staining of SA-ß-Gal has also been detected *in vitro* in late passage RPE cultures [49], [50] and *in vivo* in the RPE cells of old primate eyes [51]. In human RPE cells, an increased expression of SA-ß-Gal staining could be triggered by mild hyperoxia-mediated ROS release [52]. Furthermore, cellular senescence can also be identified by increased expression of senescence-associated biomarkers such as Apo J, CTGF, and fibronectin. All three biomarkers are inducible by oxidative stress [16], [18]. In our experiments, exposure of primary human RPE cells to CSE could lead to a significant elevation of Apo J, CTGF, and fibronectin expression. The cellular chaperone Apo J has been previously detected in the RPE of AMD donor eyes, although its role and function in the RPE is still unclear [19], [53]. In contrast, CTGF and fibronectin have been found in the Bruch's membrane, in drusen and in basal linear deposits of AMD eyes [20], [21], [22]. Furthermore, we could show that treatment of human RPE cells with CSE also increased the secretion of fibronectin and laminin into the culture media. Laminin is a basement membrane protein, which is involved in the formation of basal laminar deposits of the ageing macula [24]. Both laminin and fibronectin have been shown to be secreted by senescent human RPE cells [23]. In the pathogenesis of AMD, it is assumed that cellular senescence and dysfunction of the RPE lead to an increased aggregation of ECM [15], [54]. Therefore, CSE-induced levels of CTGF and fibronectin represent senescence-associated changes and demonstrate increased ECM synthesis in cultured human RPE cells. A similar effect could also be observed after treatment of RPE cells with hypoxia/reoxygenation [55]. Furthermore, exposure to cigarette smoke could increase the formation of sub-RPE ECM deposits in an experimental mouse model [56], [57]. An induction of CTGF levels was previously observed during cutaneous wound healing in smoke-exposed mice [58]. Whether or not CSE is responsible for the ECM accumulation in the RPE of AMD patients awaits further investigations.

Based on these results, we conclude that cigarette smoke may be responsible for the cell loss, senescent changes, and synthesis of ECM components in primary cultured human RPE cells. Therefore, cigarette smoke may induce cellular events, which may resemble pathogenic changes in AMD. Hence, these results may provide one explanation for the adverse effects of cigarette smoke on the pathogenesis and progression of AMD.
